# Functional Analyses of Bovine Foamy Virus-Encoded miRNAs Reveal the Importance of a Defined miRNA for Virus Replication and Host–Virus Interaction

**DOI:** 10.3390/v12111250

**Published:** 2020-11-02

**Authors:** Wenhu Cao, Erik Stricker, Agnes Hotz-Wagenblatt, Anke Heit-Mondrzyk, Georgios Pougialis, Annette Hugo, Jacek Kuźmak, Magdalena Materniak-Kornas, Martin Löchelt

**Affiliations:** 1 Division Viral Transformation Mechanisms, Research Focus Infection, Inflammation and Cancer, German Cancer Research Center (Deutsches Krebsforschungszentrum, DKFZ), 69120 Heidelberg, Germany; mckf11111@gmail.com (W.C.); stricker@bcm.edu (E.S.); g.pougialis@dkfz.de (G.P.); a.hugo@dkfz.de (A.H.); 2 Core Facility Omics IT and Data Management, German Cancer Research Center (Deutsches Krebsforschungszentrum, DKFZ), 69120 Heidelberg, Germany; hotz-wagenblatt@dkfz.de (A.H.-W.); a.heit@dkfz.de (A.H.-M.); 3 Department of Biochemistry, National Veterinary Research Institute, 24–100 Pulawy, Poland; jkuzmak@piwet.pulawy.pl (J.K.); magdalena.materniak@piwet.pulawy.pl (M.M.-K.)

**Keywords:** bovine foamy virus, spumaretrovirus, miRNA expression, virus-host-interaction, miRNA target gene identification, innate immunity, ANKRD17, Bif1 (SH3GLB1), replication in vitro

## Abstract

In addition to regulatory or accessory proteins, some complex retroviruses gain a repertoire of micro-RNAs (miRNAs) to regulate and control virus–host interactions for efficient replication and spread. In particular, bovine and simian foamy viruses (BFV and SFV) have recently been shown to express a diverse set of RNA polymerase III-directed miRNAs, some with a unique primary miRNA double-hairpin, dumbbell-shaped structure not known in other viruses or organisms. While the mechanisms of expression and structural requirements have been studied, the functional importance of these miRNAs is still far from understood. Here, we describe the in silico identification of BFV miRNA targets and the subsequent experimental validation of bovine Ankyrin Repeat Domain 17 (ANKRD17) and Bax-interacting factor 1 (Bif1) target genes in vitro and, finally, the suppression of ANKRD17 downstream genes in the affected pathway. Deletion of the entire miRNA cassette in the non-coding part of the U3 region of the long terminal repeats attenuated replication of corresponding BFV mutants in bovine cells. This repression can be almost completely trans-complemented by the most abundant miRNA BF2-5p having the best scores for predicted and validated BFV miRNA target genes. Deletion of the miRNA cassette does not grossly affect particle release and overall particle composition.

## 1. Introduction

In addition to the structural genes, viruses often encode proteins or non-protein regulators of viral replication to modulate the interplay with their hosts. While regulatory proteins are common in many viruses, several DNA viruses additionally encode miRNAs as part of RNA polymerase II or III (RNA Pol II and III) primary transcripts that are common viral factors in the virus–host interaction [1]. On the other side, cellular miRNAs often target essential viral pathways in order to inhibit virus replication, but in rare cases, cellular miRNAs may even fulfill critical functions, for instance, during hepatitis C virus replication [2,3]. By acquisition of counter-acting miRNAs or proteins interfering with host-encoded miRNA expression, maturation, and function, viruses often inhibit this restriction (reviewed in Skalsky et al., 2010; [4]).

In retroviruses, miRNAs are rare compared to regulatory and accessory proteins [1]. There is clear evidence of avian leucosis virus subgroup J (ALV-J) miRNA expression via RNA Pol II [5]. The issue whether HIV expresses miRNAs is controversial: HIV miRNAs are expressed at very low levels and these short RNAs often display deviant features such as their size, as discussed by Balasubramaniam [6]. In clear contrast, canonical miRNAs have been detected at considerable to even very high levels in cells infected with bovine leukemia virus (BLV, [7]), bovine foamy virus (BFV, [8]), and simian foamy virus of African green monkeys (SFV_agm_, [9]) and Japanese macaques (SFV_cae_, [10]).

Foamy viruses (FV), or spumaretroviruses, comprise five genera of the Spumaretrovirinae [11]. FVs are characterized by several unique features in their molecular biology and replication strategy compared to the Orthoretrovirinae encompassing the remaining majority of the retrovirus family including the deltaretrovirus BLV [11,12,13,14,15]. Due to their apathogenicity, broad range of susceptible host cells, and other features, FVs are promising candidates for novel virus vectors [16]. In contrast, their capacity to cross species barriers leading to zoonotic infections of humans by simian FVs and their presence in the human food chain are important for human and animal health and may serve as a model for other viruses with similar features [17]. Here, BFV is of particular interest as a co-pathogen in livestock animals, which may or may not modulate the disease potential of other known pathogens [13,18]. 

Expression of the primary miRNAs (pri-miRNAs) of BLV, BFV, SFV_agm_, and SFV_cae_ is directed by unique short transcripts generated by RNA Pol III [7,8,9,10]. While in BLV the pri-miRNAs consist of individual, single stem-loop structures, the single pri-miRNA species of BFV and some of those from SFV_agm_ and SFV_cae_ consist of two closely spaced stem-loops with an overall dumbbell structure [7,8,9,10,13]. In SFV_agm_ and SFV_cae_, additional pri-miRNAs may be similar to those of BLV consisting of only a single stem-loop [9,10,13]. The single- and double stem-loop RNA Pol III pri-miRNAs of FVs as summarized by Materniak et al., 2019 [13] may be the end-product of complex evolutionary processes that may have been primarily shaped by the limited genetic coding capacity of retro- and spumaviral genomes. In addition, protection of the genomic, full-length RNA from the miRNA processing machinery may have led to the acquisition or emergence of RNA Pol III-directed short pri-miRNAs as discussed by Cullen [2].

To our knowledge, an RNA Pol III-driven dumbbell-shaped pri-miRNA is a unique feature of BFV, SFV_agm_, and SFV_cae_ with unambiguous experimental data on the expression (abundance), processing, and potential utilization for translational purposes [8,9,10,13,19]. For both SFVs, target gene prediction has been performed and some of the targets have been validated based on reporter constructs in heterologous cells [9,10]. Since BFV has only a single dumbbell-shaped pri-miRNA and correspondingly only a single expression cassette, it appears to be a comparably simple and easy to engineer model compared to SFV_agm_ and SFV_cae_ with a less defined mix of single- and double-stem-loop structures [8,9,10,13].

Here, we describe BFV miRNA target gene prediction studies and identification of the importance of the high-abundance BFV BF2-5p miRNA for BFV replication. Bovine ANKRD17 [20,21,22,23] and Bif1 [24,25] were shown to be BF2-5p target genes in vitro with respect to moderately decreased RNA and strongly reduced protein steady state levels, similar to the action of most other miRNAs [3,26]. Besides functions in several cellular pathways, ANKRD17 is an upstream regulator of innate immune signaling [20,21,22,23] and corresponding changes were detected here. Infectivity of an infectious BFV clone devoid of the miRNA cassette displayed attenuated replication in vitro, which could be trans-complemented by the BF2-5p miRNA. Deletion of the complete miRNA expression cassette had no obvious effects on Gag and Pol expression, processing and particle release and composition.

## 2. Materials and Methods

### 2.1. Cell Culture and DNA and miRNA Transfection Techniques

HEK293T human epithelial kidney cell line (ATCC CRL-3216; Manassas, VA, USA), baby hamster kidney cell line 21 (BHK, ATCC CCL-10) and the bovine macrophage cell line BoMac [27] were used for transfection assays. These cells were grown in Dulbecco’s modified Eagle medium (DMEM, Sigma, Steinheim, Germany) supplemented with 10% fetal calf serum (Biochrome, Berlin, Germnay) and 1% penicillin–streptomycin (GIBCO, New York, NY, USA). Bovine Madin–Darby bovine kidney (MDBK, ATCC CCL-22) cells and the MDBK-derived BFV Tas reporter MICL cells, carrying an EGFP gene upstream of the BFV LTR promoter were grown in DMEM as above [28]. All cells used were routinely checked for the absence of mycoplasma and cell identity by multiplex analyses (GATC, Konstanz, Germany).

The day before transfection, cells were seeded at a density of 20–30%. The next day, cells were transfected with plasmid DNA by using the lipofectamine LTX reagent (Thermo Fisher Scientific, Darmstadt, Germany) according to the manufacturer’s instruction or polyethylenimine (PEI, Polysciences Inc., Warrington, PA, USA) [8,28]. After incubation for 12 h at 37 °C and 5% CO_2_, the medium was replaced by fresh medium and the cells were incubated for an additional 36 h before lysis and luciferase reporter assays.

A molecular mimic and the corresponding scrambled sequence miRNA for BFV BF2-5p miRNA of BFV were purchased from Invitrogen/Thermo Fisher Scientific and transfected using lipofectamine LTX reagent and 25 pmol miRNA per well of a 6-well plate. The miRNA mimic and control are small, chemically modified double-stranded RNAs while the miRNA inhibitor is a small, chemically modified single-stranded RNA designed to specifically bind to and inhibit BF2-5p (for details see: https://www.thermofisher.com/de/de/home/life-science/epigenetics-noncoding-rna-research/mirna-analysis/mirna-mimics-inhibitors/mirvana-mimics-inhibitors.html, accessed on 1.12.2017).

### 2.2. Molecular Cloning

All BFV miRNA expression plasmids were based on the RNA Pol-II and -III promoter-deficient basal vector pGEM3Z (Promega, Mannheim, Germany) and the different dual reporter constructs have been described previously [19]. To construct additional psiCHECK2-based reporter plasmids for miRNA function, genomic sequences were amplified using the high-fidelity Phusion DNA polymerase (New England Biolabs, Frankfurt, Germany), 0.5 µmol of the primers, and 200 ng target DNA from wt BFV-Riems [29] or bovine MDBK cells under the following conditions: Initial denaturation at 98 °C for 30 s, then 30 cycles of amplification (98 °C for 10 s, 55 °C for 30 s, 72 °C for 1 min), final extension at 72 °C for 10 min as described previously [19]. Reporter constructs for bovine ANKRD17 (NM_001192181.2) and Bif1 (NM_001077993.2) were confirmed by DNA sequencing and each encompassed about 400 nt 3′ UTR sequences.

To conduct reverse genetic experiments, cloned BFV genomes selected for high titer cell-free transmission in bovine MDBK cells [28,30] were further optimized by replacing the U3 region of the 5′LTR by the strong and constitutively active CMV immediate early (IE) promoter using standard cloning strategies as described previously for feline FV [31]. BFV genomes lacking the complete BFV miRNA cassette were obtained by fusion PCR mutagenesis as described [32,33], utilizing the following primer pair 1 (fw-NheI-ENV; 5′-GTTAGTCATCGGAATATTGAGATGGCTAGCGGTG-GGACGCCGG-3′ and rev-AscI-U3; 5′-CAGATCTCAGGCGCGCCGGTTCCTTATTGAGATGTC-TTCG-3′) and primer pair 2 (fw-AscI-AclI-U3, 5′-AATAAGGAACCGGCGCGCCTGAGAT-CTGTGTGTGACTACATTGAACGTTGATGTATAACTAGAAGAATAAGATTAAG-3′ and rev-ApaI-U5; 5′-GACTCACTATAGGGCGAATTGG-GCCCTTGTTGTGACCTTCTCC-3′; all from Sigma). The final amplicon was generated in an independent PCR using both initial amplicons and primers fw-NheI-ENV and rev-ApaI-U5 and inserted using the unique NheI and ApaI sites. Mutagenesis resulted in the deletion of a 143 bp region encompassing the BFV miRNA cassette and insertion of unique AscI and AclI restriction sites conserving 5/15 bps. Replacement of the U3 region of the 5′ LTR by CMV-IE promoter sequences avoided recombination between the still intact miRNA cassette in the 5′ LTR and the 3′LTR in which the miRNA cassette sequences had been deleted. In BFV, miRNA deletion mutants carrying the authentic U3 sequence in the 5′LTR such as recombination events were in fact observed and enriched during extended passaging of such clones [34].

### 2.3. Dual Luciferase Reporter Assays

A total of 50 ng of each control or BFV dual-luciferase reporter (DLR) target plasmids was co-transfected with 500 ng of a pGEM3Z-based BFV-miRNA expression vector using Lipofectamine LTX or PEI into 24-well plates containing 10^5^ BHK, HEK293T, or MDBK cells per well, which had been plated the previous day as previously described [14]. All transfections for DLR assays were done in duplicate and repeated at least once. At 48 h after transfection, lysates were harvested and renilla and firefly luciferase (Rluc and hluc) activities were measured in triplicate using a dual-luciferase reporter assay system (Promega, Walldorf, Germany) on a TD-20/20 luminometer (Turner Designs, San Jose, CA, USA). The ratio of the activities of RLuc linked to the BFV miRNA target sequences and hluc serving as an internal control was determined for each sample and normalized to the ratio for the parental psiCHECK2 vector.

### 2.4. Target Gene Quantification

Reverse transcription was performed using the QuantiTect Reverse Transcription Kit (Qiagen, Hilden, Germany). In brief, total RNA was extracted by TRIzol reagent as recommended by the supplier (Thermo Fisher Scientific). Then, 1 µL Quantiscript Reverse Transcriptase, 4 µL Quantiscript RT Buffer, and 1 µL RT Primer Mix were combined with 500 ng total RNA and incubated for 15 min at 42 °C. Samples were finally incubated for 3 min at 95 °C to inactivate the reverse transcriptase. Real-time PCR was performed by using the QuantiTect SYBR Green PCR Kit and LightCycler 480 Instrument I, and the reaction conditions were as follows: 95 °C pre-incubation and 40 cycles of 10 s at 95 °C, 40 s at 60 °C, and 1 min at 72 °C. The following primer pairs were used for bovine Ankrd17: fwd, 5′-GCGCGGTACCTATGGAGAAGGCGACGGTTCC-3′; rev, 5′-GCGCGCGGC-CGCTCAGCCAGCTGGTTCATATGCA-3′; bif1: fwd, 5′-TCCTTCCAACCTCAGTGACCTT-3′; rev, 5′-TCATAGAGAACCCTGGCCTTTC-3′; IFN-beta: fwd, 5′-AAACTCATGAGCAGTCTGCA-3′; rev, 5′-AAACTCATGAGCAGTCTGCA-3′; NF-kappaB: fwd, 5′-GAAATTCCTGATCCAGACAAAAAC-3′; rev, 5′-ATCACTTCAATGGCCTCTGTGTAG-3′; GAPDH: fwd, 5′-CGAGATCCCTCCAAAA-TCAA-3′; rev, 5′-TTCACACCCATGACGAACAT-3′. Relative gene abundances were determined using the ΔΔ*CT* method with GAPDH as the reference [35].

### 2.5. In Silico Target Gene Prediction and Statistical Analyses

BFV miRNA sequences were used as inputs for two online target prediction databases: miRanda (http://www.microrna.org/microrna/home.do, accessed on 10 August 2017) and TargetScan (http://www.targetscan.org, accessed on 10.8.2017). In order to increase the identification of meaningful miRNA target candidates, the use of more than one tool is recommended [36,37]. Using the miRanda and TargetScan miRNA prediction algorithms, the bovine *Bos tauris* genome was scanned for complementarity to the three stable BFV miRNAs miR-BF1-5p, miR-BF2-5p, and miR-BF1–3p [8]. TargetScan seems to be a more robust tool, because it enables a more complete search at the isoform level and penalizes the less conserved interactions, and its databases are the most up-to-date. For these reasons, TargetScan can predict the interactions with a higher probability of being biologically validated than the other tools. However, the algorithms used by miRanda (mirSVR score) can complement these prediction studies, as it takes additional biological parameters into consideration that remain interesting to verify. Only those potential target genes that were detected by both algorithms were selected and analyzed further. To reduce the number of hits to biologically meaningful and relevant target genes, the search was restricted to the 3′-untranslated regions (UTR) of the bovine target genes in line with general observation that miRNA target sites are mostly, but not exclusively, located in this region [3,38].

Data are expressed as the means ± standard deviations of the results of at least two independent experiments, in which each assay was performed in triplicate. Statistical analyses were conducted using the Welch’s and Student’s *T*-test using in-house and online resources (graphpad.com, accessed on 5 February 2018). *p* values of <0.05 were considered to be statistically significant. 

### 2.6. Virological Methods

High titer cell-free transmitted BFV variants with and without the miRNA expression cassette were transfected into HEK293T cells and supernatants harvested two to four days after transfection as indicated. BFV titrations were done using serially diluted cell-free supernatants under spreading infection conditions with MICL reporter cells seeded at low cell density to allow repeated rounds of virus replication and multiplication. BFV infectivity was determined by quantification of viral foci and is expressed as focus-forming units/mL (FFU/mL) as described [28]. BFV infections with wt and miRNA-deficient BFV for reporter assays and expression studies were at multiplicities of infection (MOI) of 0.1 to 0.05, except otherwise stated. BFV particles released into the cell culture supernatant were enriched by sedimentation through a 20% (*w*/*v*) sucrose cushion in an SW41 rotor at 28,000 rpm for 2 h at 4 °C as described previously [28].

### 2.7. Protein Analysis by Immunoblotting

Cells and enriched virus particles were lysed in 1% SDS with protease inhibitor, mixed with 5× Reducing Sample Loading Buffer and denatured by heating at 95 °C for 5 min. Samples were loaded onto Novex 4–12% Bis-Tris polyacrylamide gradient gels run in Bolt Mini gel chambers in 1× MOPS buffer (Thermo Fisher Scientific) at 120 V for about 120 min. Proteins were transferred onto nitrocellulose membranes at 15 V for 1 hour in a Bolt Mini blotting chamber. Blots were further processed, and specifically bound antibodies were detected as described [28]. For detection of bovine ANKRD17 and Bif1, cross-reactive antisera PA5-46799 and PA5-15278 (Thermo Fisher Scientific), respectively, were used in addition to a polyclonal rabbit antiserum directed against human ANKRD17 kindly provided by Thomas Kufer, University of Hohenheim, Germany [20]. To detect BFV proteins, a rabbit anti Gag antiserum [28], a cross-reactive rabbit anti PFV integrase (IN) antiserum [39] (kindly provided by Dirk Lindemann, Dresden), and a serum pool from BFV-infected cattle [40] were used. Densitometric analyses were carried out using ImageJ software.

## 3. Results

### 3.1. In Silico Target Prediction and Ranking

Using the two online miRNA target site prediction algorithms “miRanda–mirSVR” and “TargetScan”, the bovine *Bos tauris* genome was scanned for complementarity to the three stable BFV miRNAs miR-BF1-5p, miR-BF2-5p, and miR-BF1-3p [8]. Using the criteria based on the mean value of both algorithms as defined in Section 2.5, potential target genes for BF2-5p miRNA had scores between 100–95 out of 100 (Table 1). BF1-5p miRNA had combined scores of 80 out of 100 and less (52 out of 100 for hit #20), while scores for the 20 best hits for BF1-3p were even lower.

### 3.2. Importance of BFV miRNA Expression for Overall BFV Replication

The parental BFV Riems isolate used here was exclusively grown on primary bovine cells [41]. Recently, molecular clones of the wt BFV Riems and a BFV variant selected in vitro for a high titer (HT) replication and cell-free transmission phenotype in bovine MDBK cells were established and designated as pBFV-Riems34 and pBFV-MDBK-24 [28]. In each of these clones, the U3 promoter in the 5′LTR was replaced here by the highly efficient and constitutively active Cytomegalovirus immediate early (CMV-IE) promoter using standard cloning techniques yielding clones pCMV-BFV-Riems34 and pCMV-BFV-MDBK-24. Next, the complete miRNA cassette [8,19] was deleted by fusion PCR mutagenesis yielding clones pCMV-BFV-Riems-∆miRNA and pCMV-BFV-MDBK-∆miRNA.

**Figure 1:**
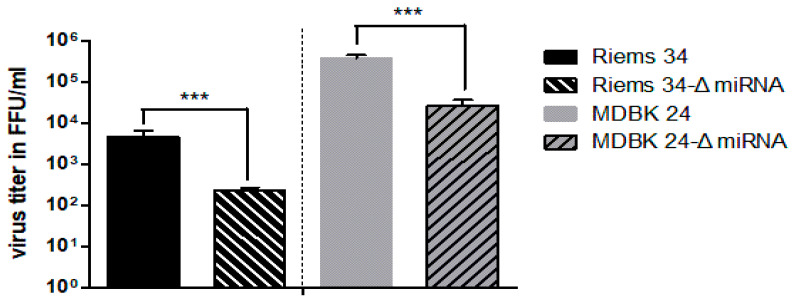
Deletion of the miRNA cassette results in reduced infectivity in bovine MICL reporter cells. Bar diagram of the virus titers (FFU/mL) expressed as number of GFP-positive focus forming units (FFU) from three independent experiments. Low-density MICL cells were infected with serially diluted wt virus (Riem 34 and MDBK 24) and the corresponding miRNA cassette deletion mutants (Riems 34-ΔmiRNA and MDBK 24-ΔmiRNA) originating from transfected HEK293T cells. Five days p.i., viral titers were determined by fluorescence microscopy of GFP-positive cell foci due to the BFV infection-mediated induction of GFP expression. Error bars are given and differences between titers of the wt and the ΔmiRNA mutant virus were analyzed by the Welch’s *t*-test and found to be highly significant (***, *p* < 0.001).

To determine whether the BFV miRNAs have detectable effects on BFV replication in in vitro cultivated bovine cells, all four CMV-IE-based BFV genomes (wt pCMV-BFV-Riems 34, wt pCMV-BFV-Riems 34-ΔmiRNA, high titer pCMV-BFV-MDBK 24, and high titer pCMV-BFV-MDBK 24-ΔmiRNA) were transfected into HEK293T cells since transfection of the approx. 15 kbp large plasmids is highly inefficient using MDBK target cells. Cell-free virus supernatants were harvested two days after transfection and infectious titers were determined by titration in the MDBK cell-based MICL LTR-GFP reporter and titration cells under spreading infection conditions (Figure 1 and ref. [28]). The data clearly show that spreading infection of both miRNA deletion mutants was significantly reduced (10-fold titer downregulation compared to the corresponding intact clones) in bovine MICL/MDBK cells, which are known to be proficient in many innate and intrinsic immunity pathways.

Due to the heterologous CMV-IE promoter that replaces the authentic BFV U3 promoter in all constructs used here (see Section 2.2), even the highly-cell-associated BFV Riems isolate yielded cell-free infectivity [30,42].

### 3.3. Deletion of the miRNA Cassette Does Not Inhibit Particle Release and Gag and Pol Expression, Processing and Packaging

**Figure 2:**
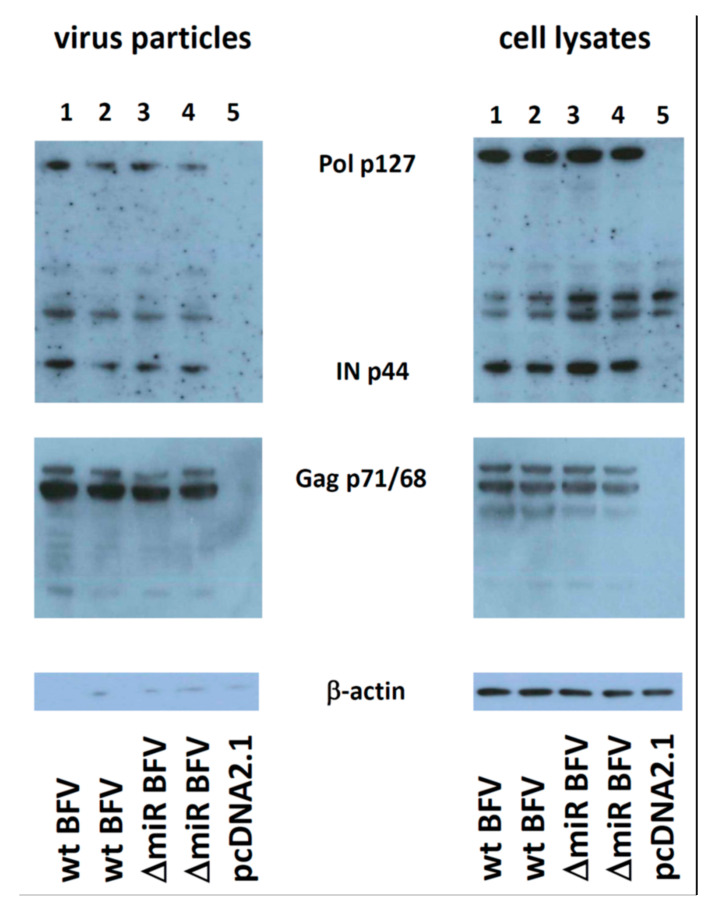
Deletion of the miRNA cassette neither affects BFV Gag and Pol expression and processing nor BFV particle release and composition in transfected and MDBK-co-cultured HEK293T cells. Each two sub-clones of plasmids encoding high titer pCMV-BFV-MDBK 24 (wt BFV) and pCMV-BFV-MDBK 24-ΔmiRNA (ΔmiR BFV) and the pcDNA2.1 control plasmid were transfected into HEK293T cells together with an EGFP expression plasmid using the PEI method. Two days later, MDBK cells were added and three days later, cells were harvested for protein analyses. Cell culture supernatants were used to enrich BFV particles by sucrose cushion sedimentation. Each 15 µg of cell lysates and regular aliquots of enriched particles were subjected to immunoblotting (as given above the panels) using a cross-reactive rabbit anti PFV IN antiserum [39] (top panels) and a serum pool from BFV-infected cattle (middle panels). The BFV-specific proteins detected are labeled between the panels. A directly conjugated antibody against β-actin served as the loading control (bottom panels).

We next addressed the question whether deletion of the miRNA cassette alters BFV Gag and Pol expression and particle release and composition. For this purpose, each two sub-clones of high titer pCMV-BFV-MDBK 24 and pCMV-BFV-MDBK 24-ΔmiRNA genomes were transfected into HEK293T cells together with an EGFP expression plasmid confirming similar transfection efficacies. After two days, fully permissive MDBK cells were added to enhance virus yield and three days later, cells were harvested for protein analyses. Cell culture supernatants were used to enrich BFV particles by sucrose cushion sedimentation and to infect low-density MICL target cells at a MOI of 0.1. Five days after MICL cell infection and at a comparable degree of infection, cells and supernatants were again harvested for BFV protein analyses. In the transfected and co-cultured cells, HEK293T/MDBK cells, and the corresponding particles (Figure 2), BFV p71 Gag and p127 Pol, precursor protein expression and processing into p68 Gag and the mature p44 IN were comparable between the BFV variants with and without the miRNA cassette. Preparations of released BFV particles contained both cleaved and uncleaved Gag and Pol proteins at comparable concentrations. The BFV-infected MICL cells and the corresponding BFV particle preparations harvested five days p.i. gained results similar to those from the transfected and co-cultured HEK293T and MDBK cells (Figure 2 and Supplementary Figure S1A). However, under the spreading infection conditions in the infected MICL cell cultures, the two independent miRNA-deficient BFV clones showed attenuated replication, which led to slightly lower protein levels in infected cell lysates and released particle preparations. However, Gag and Pol protein processing were comparable. Since FV Gag and Pol protein processing are considered to depend on proper particle formation and Pol protein encapsidation via packaged RNA genomes [43], we conclude that deletion of the BFV miRNA cassette did not detectably affect these processes under either condition. When transfected HEK293T cells were not co-cultivated with MDBK cells, but analyzed four days after transfection, similar data on overall particle release were obtained (Supplementary Figure S1B). All experiments contained duplicates and were done once.

### 3.4. Importance of the BF2-5p miRNA for BFV Replication

**Figure 3:**
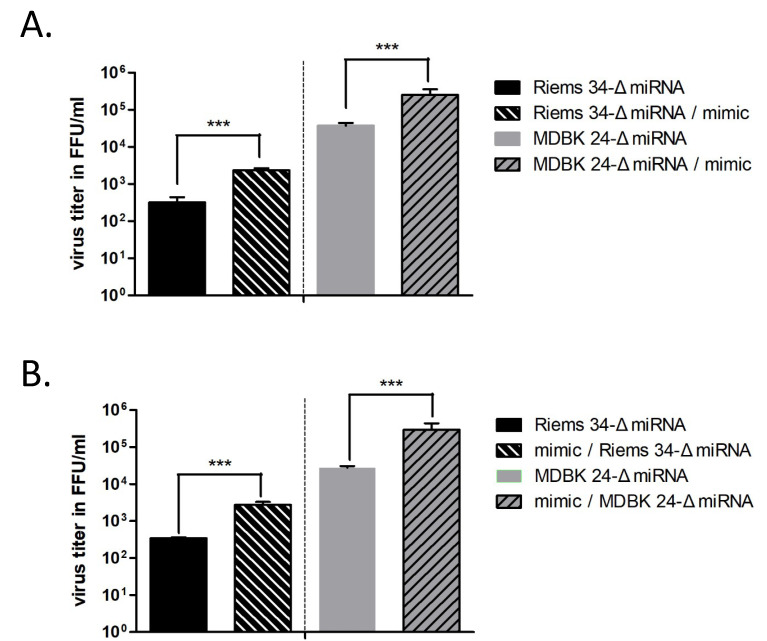
Trans-complementation of miRNA-deficient BFV with miR-BF2-5p restores BFV titers in bovine MICL cells. Bar diagrams of the virus titers (FFU/mL) and the number of GFP-positive focus forming units from three independent experiments. (**A**) MICL cells were infected first with serially diluted miRNA cassette deletion mutants (BFV-Riems 34-ΔmiRNA and BFV-MDBK 24-ΔmiRNA). Six h after infection, the corresponding miR-BF2-5p mimic and negative control were transfected. (**B**) MICL cells were transfected first with the miR-BF2-5p mimic and negative control. Six h after transfection, the cell were infected with serially diluted miRNA cassette deletion mutants (BFV-Riems 34-ΔmiRNA and BFV-MDBK 24-ΔmiRNA). At 4 day p.i., viral titers were determined in both experiments by fluorescence microscopy. Error bars for the titers determined are given and differences between titers of the viruses trans-complemented with the BF2-5p mimic and control miRNA were analyzed by the Welch’s *t*-test (***, *p* < 0.001).

**Figure 4:**
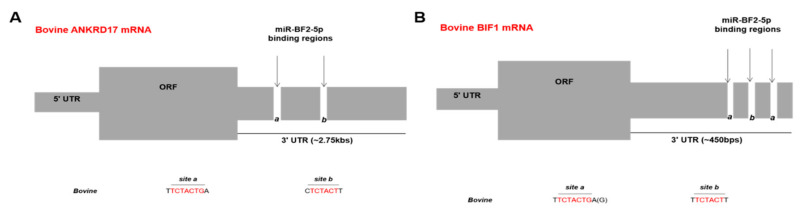
MiR-BF2-5p binding site prediction within ANKRD17 (panel **A**) and Bif1 (panel **B**). The overall bovine ANKRD17 and Bif1 genes consist of 5’UTR, the open reading frame (ORF), and the 3′UTR. Based on sequence complementarity analysis, two possible binding sites (a and b) were identified in the 3′UTR of ANKRD17 starting 85 and 448 nt downstream of the stop codon (**A**), the sequences which are complementary to the seed sequence of miR-BF2-5p, are shown in red. For Bif1 (**B**), three possible binding sites (a-b-a) were found in the 3′UTR, starting 217, 245, and 284 nt downstream of the stop codon, the sequences which are complementary to the seed sequence of miR-BF2-5p are shown in red.

Since miR-BF2-5p constitutes the most abundant miRNA in BFV-infected cells [8] and bioinformatics revealed very high target gene identification scores, we propose that this miRNA has a major impact on the BFV life cycle and virus–host interactions. We thus determined whether the differences in titers in bovine MICL target cells were due to the loss of miRNA expression or whether it is a secondary effect unrelated to the miRNAs but due to the unintended deletion of other important functions. For this purpose, miRNA trans-complementation studies using a mimic of the miR-BF2-5p with enhanced stability and identical sequence, together with a scrambled negative control oligonucleotide of the same length, were used in co-transfection studies as described [19] To additionally analyze whether the miRNAs function early or late during BFV infection, MICL cells were first infected with serial dilutions of the cell culture supernatants from pCMV-BFV-Riems 34-ΔmiRNA and high titer cell-free pCMV-BFV-MDBK 24-ΔmiRNA-transfected HEK293T cells. Six h after infection with serially diluted supernatants of the different BFV variants, cells were transfected with the miR-BF2-5p mimic and a negative control miRNA (Figure 3A). Alternatively, MICL cells were first transfected with the miR-BF2-5p mimic and the control miRNA. Approximately 6 h after transfection, the cells were infected with serial dilutions of virus supernatants from pCMV-BFV-Riems 34-ΔmiRNA and high titer cell-free pCMV-BFV-MDBK 24-ΔmiRNA-transfected HEK293T cells (Figure 3B). At four days p.i., the MICL reporter cells were screened for signs of spreading infection as indicated by infection-induced GFP fluorescence. For both BFV miRNA-deficient variants, the co-transfection of the mimic BF2-5p miRNA reproducibly increased the virus titer ten-fold over the control (Figure 3A,B). Furthermore, there was no significant titer difference between the ‘infection-first’ and ‘transfection-first’ assays. These data demonstrate that miR-BF2-5p plays an important role in BFV replication, significantly increasing virus spread and infectivity when provided 6 h before and after infection. At the same concentration, an inhibitor of miR-BF2-5p that acts as a sponge to bind and neutralize its target miRNA did not show any effect on the miRNA-containing parental BFV Riems and its high titer variant [44]. This may have been due to the fact that its concentration was too low to neutralize the highly abundant BF2-5p miRNA or that we did not replenish the inhibitor over the incubation period. In addition, the specific expression strategy of the BFV miRNAs and/or the presence of substantial amounts or pri-miRNA in BFV-infected cells (see Figure 4 in reference [8]) may have negatively affected the functions of the inhibitor miRNA.

The specificity and absence of significant off-target effects of the BFV miR-BF2-5p mimic, control, and inhibitor (for details see Section 2.1) used throughout the current study were confirmed in one control experiment (with duplicates) using the two high-score target genes encoding for ANKRD17 and Bif1 (Supplementary Figure S2). Importantly, the gene-specific controls presented here do not rule out additional and potentially genome-wide off target effects as known to occur when conducting corresponding functional miRNA analyses [45].

### 3.5. In Vitro Validation of ANKRD17 and Bif1 as Direct Targets of miR-BF2-5p

Data mining of the literature of the high score BF2-5p mRNA targets and potential links to innate immunity and virus replication identified several interesting candidate genes. Among them, the human gene ANKRD17 (ankyrin repeat domain 17) is a regulator of NOD/RLR-mediated inflammatory responses and DNA replication [20], while Bif1 (Bax-interacting factor 1, also designated SH3 domain-containing GRB2-like endophilin B1, SH3GLB1 or ZBTB24) is involved in autophagy and apoptosis-induced virus elimination [24,25]. Since only a few antibodies specifically raised against bovine proteins have been available, we screened several bovine candidate proteins for cross-reactive and commercially available antisera against human or mice counterparts [44]. Based on this survey, target gene validation was performed for bovine ANKRD17 and Bif1.

For validation of ANKRD17 and Bif1 as direct targets of BFV miR-BF2-5p, in silico binding site predictions were performed. The whole mRNA sequences of bovine ANKRD17 (NM_001192181.2, NCBI) and Bif1 (NM_001077993.2, NCBI) were acquired and then the miR-BF2-5p (especially the seed sequences that are important for base-pairing with complementary sequences on the target mRNAs) were aligned with ANKRD17 and Bif1 mRNAs. Using this approach, two potential binding/target sites for miR-BF2-5p were identified inside the 3′UTR region of ANKRD17 (Figure 4A), while three potential binding/target sites for miR-BF2-5p were discovered inside the 3′UTR region of Bif1. Two of the BF2-5p miRNA binding sites inside the 3′UTR region of Bif1 were almost identical (Figure 4B, designated site a with variant flanking sequences).

To verify that miR-BF2-5p can target the predicted binding sites, the subgenomic sequence, which contains the two predicted binding sites in the ANKRD17 3′UTR and the three predicted binding sites in the 3′UTR of Bif1, were amplified via PCR, cloned into the reporter plasmid psiCHECK2, and named psiCHECK2-ANKRD17-original (A.Ori) and psiCHECK2-Bif1-original (B.Ori). The corresponding scrambled target sequence, which is no longer complementary to miR-BF2-5p, were also cloned into the reporter plasmid and named psiCHECK2-ANKRD17-scrambled (A.Scr) and psiCHECK2-Bif1-scrambled (B.Scr).

**Figure 5:**
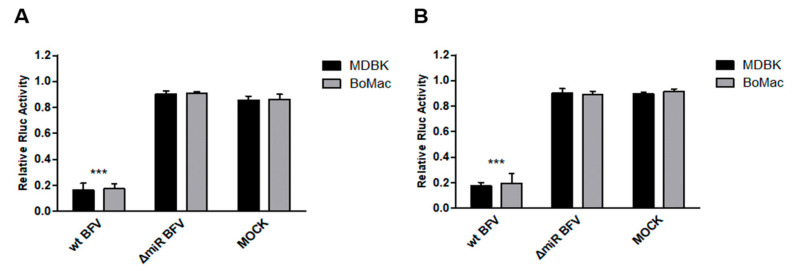
Binding efficacy of miR-BF2-5p and ANKRD17 (**A**) and Bif1 (**B**) target sequences in BFV-infected cells. MDBK and BoMac cells (as given) were infected with high-titer BFV-MDBK24 (wt BFV) and miRNA-deficient BFV-MDBK24 (ΔmiRNA) at a MOI of 0.1 or cell culture medium was added (MOCK). Two days p.i., the reporter plasmids psiCHECK2-ANKRD17-original (A.Ori, panel A) and psiCHECK2-Bif1-original (B.Ori, panel B) were transfected into the cells. At 24 h post-transfection, cell lysates were harvested and analyzed for the suppression of Rluc activity in standard DLR assays as described. Normalized luciferase data (Renilla versus firefly luciferase activity) are presented as bar diagrams of three independent experiments. Differences between treated and control groups (MOCK) were analyzed by the Welch’s *t*-test (***, *p* < 0.001).

Then, plasmids pCMV-BFV-MDBK24, pCMV-BFV-MDBK24-ΔmiRNA, and empty plasmid pCR-XL-Topo were transfected into HEK293T cells and 48 h after transfection, BFV, and mock cell culture supernatants were harvested. Each four cultures of MDBK and BoMac cells were infected with normalized amounts of virus supernatants and 2 days p.i., the reporter plasmids A.Ori (Figure 5, panel A) and B.Ori (Figure 5, panel B) were transfected into the infected cells. As the control, two cultures each were mock infected but transfected as above. Twenty-four hours post transfection, the cells were harvested for the dual luciferase reporter (DLR) assay. The normalized DLR readouts showed that in both bovine cell lines, a significant downregulation of Rluc activity in the wt BFV infection group occurred. In contrast, no changes of Rluc activity in the ΔmiRNA BFV infection group and the mock-infected sample were detectable (Figure 5). The data demonstrate that the miR-BF2-5p expressed by the wt BFV virus can target the predicted binding sites within the 3′UTR of ANKRD17 and Bif1.

**Figure 6:**
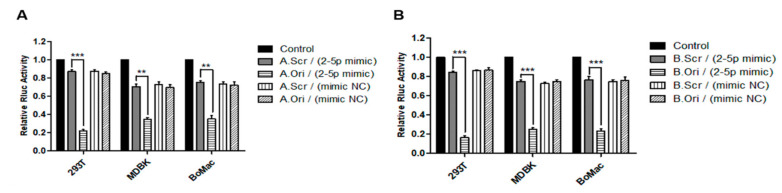
Binding efficacy of miR-BF2-5p mimic and the target sequences in the 3′UTR of bovine ANKRD17 (**A**) and Bif1 (**B**). HEK293T, MDBK, and BoMac cells were transfected with four reporter plasmid/RNA oligonucleotide combinations (letters A and B refer to the ANKRD17 and Bif1 target genes and suffixes Ori and Scr refer to original (wild-type) and scrambled (non-functional control) target sequences, respectively): A.Scr/B.Scr plus miR-BF2-5p mimic (A.Scr/B.Scr (2–5p mimic)); A.Ori/B.Ori plus miR-BF2-5p mimic (A.Ori/B.Ori (2–5p mimic)); A.Scr/B.Scr plus mimic NC (A.Scr/B.Scr (mimic NC)); A.Ori/B.Ori plus mimic NC (A.Ori/B.Ori (mimic NC)). In addition, the empty DLR report plasmid was used as control (black bars). At 24 h post transfection, cell lysates were harvested and analyzed for the suppression of Rluc activity in standard DLR assays as described. Normalized luciferase data (Renilla versus firefly luciferase activity) are presented as bar diagrams of three independent experiments. Differences between treated groups and MOCK or Control groups were analyzed by the Welch’s *t*-test (***, *p* < 0.001; **, *p* < 0.01).

To confirm that BF2-5p in fact induces suppression of target constructs in BFV-infected cells, a stable mimic of the miR-BF2-5p with enhanced stability and identical sequence together with a scrambled negative control oligonucleotide of the same length were used in co-transfection studies as described [19]. These RNA oligonucleotides together with the two ANKRD17 and Bif1 reporter plasmids (original and scrambled) were co-transfected into HEK293T, MDBK, and BoMac cells in the following four combinations for each target gene (Figure 6A,B): A.Scr/B.Scr reporter plus miR-BF2-5p mimic RNA oligonucleotide; A.Ori/B.Ori reporter plus miR-BF2-5p mimic oligonucleotide; A.Scr/B.Scr reporter plus mimic negative control (mimic NC) oligonucleotide; and A.Ori/B.Ori plus mimic NC. At 24 h post-transfection, cells were harvested for DLR analyses. Transfection of three different cell lines showed significant Rluc activity suppression only for the combination of A.Ori/B.Ori plus miR-BF2-5p mimic, while all other combinations did not result in target gene suppression. These data demonstrate that the miR-BF2-5p mimic specifically targets the predicted binding sites within the 3′UTR of the bovine ANKRD17 and Bif1 genes, leading to their specific suppression (Figure 6).

**Figure 7:**
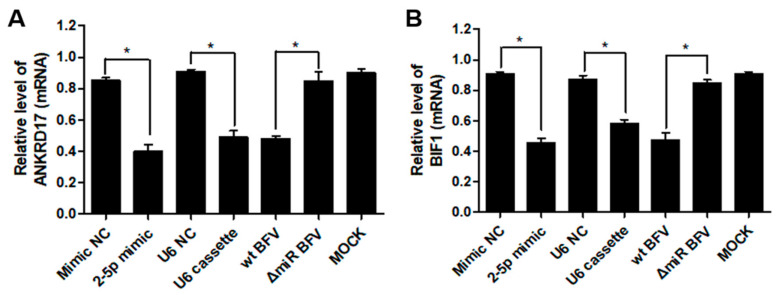
Validation of ANKRD17 (**A**) and Bif1 (**B**) mRNAs as direct targets of miR-BF2-5p. MDBK cells were transfected with miR-BF2-5p mimic and mimic NC (negative control), or U6 promoter driven cassette and U6 promoter empty plasmid (NC), or infected with wt BFV-MDBK24 and ΔmiRNA BFV-MDBK24. After three days, transfected, infected, and untreated cells (MOCK) were harvested and RNA was extracted for qRT-PCR analysis. Expression data normalized relative to GAPDH RNA are shown as bar diagrams of three independent experiments. Differences between treated groups and MOCK or negative control groups were analyzed by the Welch’s *t*-test (*, *p* < 0.05).

To determine whether miR-BF2-5p can suppress the steady state mRNA levels of bovine ANKRD17 (Figure 7A) and Bif1 (Figure 7B), the mimic, the U6 promoter driven BFV miRNA expression vector pBS-U6-BFVmiRNA (4G, [19]) and the corresponding controls were transfected into MDBK cells. In parallel, a second group of MDBK cells was infected with wt BFV-MDBK24 and ΔmiRNA BFV-MDBK24 (MOI of about 0.1). After three days, cells from both experiments (transfections and infections) were harvested and total RNAs extracted for qRT-PCR analyses. The results indicate a moderate but significant suppression of the corresponding bovine mRNA levels for ANKRD17 and Bif1 only in MDBK cells expressing or containing miR-BF2-5p (mimic, U6-cassette, wt BFV infected) (Figure 7). The findings demonstrate that miR-BF2-5p specifically targets ANKRD17 and Bif1 mRNAs and causes moderate but significant suppressions of the steady state mRNA levels. The transfected miRNA species did not lead to gross changes of the transfected cells [44].

**Figure 8:**
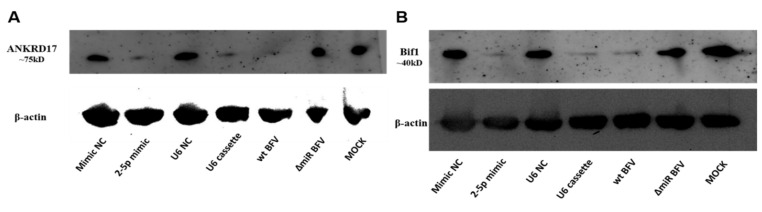
Validation of steady-state protein levels of ANKRD17 (**A**) and Bif1 (**B**) as direct targets of miR-BF2-5p. MDBK cells were transfected with miR-BF2-5p mimic and mimic NC (negative control), or U6 promoter driven cassette and U6 promoter empty plasmid (NC), or infected with wt BFV-MDBK24 and ΔmiRNA BFV-MDBK24. After three days, the transfected and infected cells were harvested and protein was extracted for western blotting. The expression of β-actin was monitored as a control for proper protein loading. In this blot representing one out of two experiments, the 75 kDa splice variant of bovine ANKRD17 and the full-length 40 kDa Bif1 proteins were specifically detected using antibodies as described in Section 2.7.

To analyze whether miR-BF2-5p also suppresses steady-state ANKRD17 (Figure 8A) and Bif1 (Figure 8B) protein levels, the mimic, the U6 promoter driven cassette expression vector pBS-U6-BFVmiRNA (4G), and the corresponding controls were transfected into MDBK cells. In parallel, a second group of MDBK cells was infected with wt BFV-MDBK24 and ΔmiRNA BFV-MDBK24 at a MOI of about 0.1. After three days, total cellular protein lysates were harvested from all samples and identical amounts of protein were subjected to western blot analyses. The results showed, in both experiments (transfections and infections), a strong decrease in the ANKRD17 and Bif1 protein levels (Figure 8A,B) in MDBK cells expressing or containing miR-BF2-5p (mimic, U6-cassette, wt BFV infected). Densitometric analyses revealed about 5-fold decreases of ANKRD17 by co-transfection of the BF2-5p mimic and the U6-based miRNA expression construct and a 20-fold decreases by superinfection with wt BFV compared to the corresponding controls. Bif1 levels were reduced between 12- to 17-fold by the corresponding BFV miRNA treatment while a two-fold decline was seen in this experiment by infection with the miRNA-deficient BFV variant. The experiment demonstrates via different approaches that miR-BF2-5p significantly and specifically targets and decreases steady state protein levels of the 75kD ANKRD17 splice variant and the intact, full-length Bif1 protein. Consistent with the function of miRNAs mainly at the translational level [3,26], target protein levels were much more affected than those of the corresponding mRNAs (see Figure 7).

Overall, the various complementing analyses fully validate bovine ANKRD17 and Bif1 as direct targets of the BFV miR-BF2-5p.

### 3.6. MiR-BF2-5p Suppresses Expression of Innate Immunity Genes as Predicted Downstream Targets of ANKRD17

**Figure 9:**
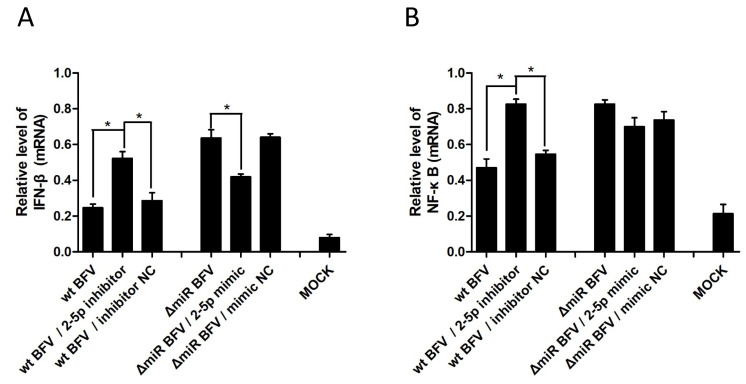
Suppression of pro-inflammatory signaling via IFN-β (**A**) and NF-κB by (**B**) miR-BF2-5p. BoMac cells were infected with wt BFV-MDBK24 and 2 d p.i. transfected with miR-BF2-5p inhibitor and inhibitor negative control (NC) RNA oligonucleotides. In parallel, another group of BoMac cells were infected with ΔmiRNA BFV-MDBK24 and 2 d p.i. transfected with miR-BF2-5p mimic and mimic NC RNA oligonucleotides. At 48 h after transfection, cells were harvested and total RNA was extracted for qRT-PCR analyses, testing the expression level of IFN-β and NF-κB, respectively. Expression data normalized relative to GAPDH RNA are shown as bar diagrams of three independent experiments. For comparison, the relative and GAPDH-normalized expression levels of IFN-β and NF-κB of untreated BoMac cells are given (MOCK). Differences between treated groups and MOCK or negative control groups were analyzed by the Welch’s *T*-test (*, *p* < 0.05).

Based on the data that ANKRD17 is an upstream regulator in innate immunity [20,21,22,23], the role of miR-BF2-5p in host cell innate immunity, specifically in pro-inflammatory signaling via interferon-beta (IFN-β) and nuclear factor kappaB (NF-κB), was evaluated. BoMac cells were infected with wt BFV-MDBK24 and ΔmiRNA BFV-MDBK24 at a MOI of about 0.1 and 2 d p.i., the miR-BF2-5p inhibitor RNA oligonucleotide and negative control (inhibitor NC) were transfected into the cells infected with intact BFV-MDBK24, in parallel, the miR-BF2-5p mimic and negative control (mimic NC) were transfected into the cells infected with miRNA-deficient BFV-MDBK24. Cells were harvested and total RNA was extracted for qRT-PCR analyses 48 h after transfection. The results showed a significant upregulation of IFN-β and NF-κB mRNA levels (Figure 9A,B) for wt and miRNA-deficient BFV-infected and transfected cells compared to mock-treated cells. In wt BFV-infected BoMac cells, the inhibitor of miR-BF2-5p led to a further increase in IFN-β and NF-κB mRNA levels, which was not seen when the corresponding control miRNA was co-transfected. Furthermore, in ΔmiRNA BFV-MDBK24-infected cells, steady state mRNA levels for IFN-β and NF-κB were further enhanced. Co-transfection of the miR-BF2-5p mimic resulted in decreased IFN-β levels while NF-κB levels were not suppressed. Again, transfection of the different miRNAs did not detectably affect cell viability or led to aberrant changes of the analyzed target mRNAs. The results presented here suggest that BFV miR-BF2-5p suppresses cellular pro-inflammatory signaling by IFN-β and NF-κB, which had been increased by BFV infection. The lack of complementarity of the miRNA mimic and inhibitor studies in panel B may be due to different efficacies of the transfected miRNAs or different kinetics of gene activation versus miRNA-induced suppression (see also Section 4).

### 3.7. Kinetics of ANKRD17 and Bif1 Suppression during Productive BFV Infection of MDBK and BoMac Cells

**Figure 10:**
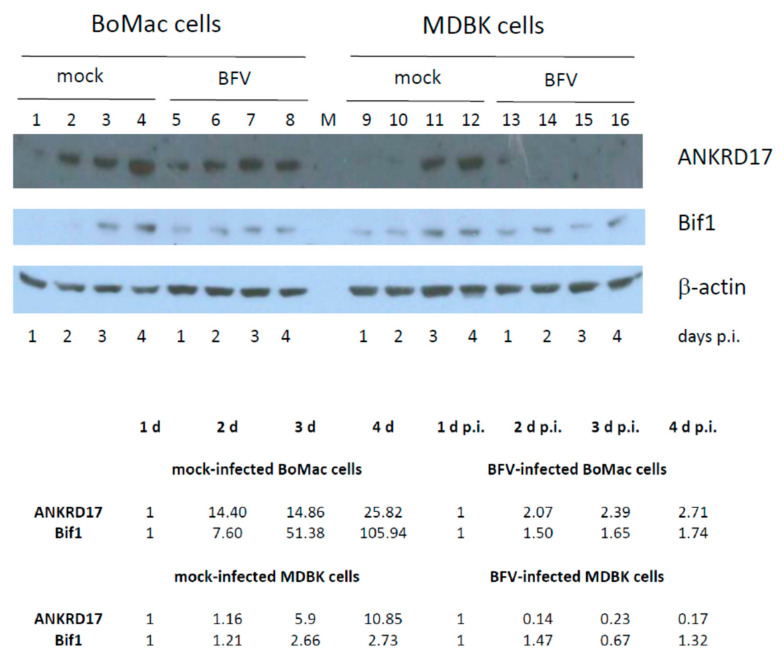
Kinetics of ANKRD17 and Bif1 steady state levels in BFV-infected and mock-infected BoMac (lanes 1 to 8) and MDBK cells (lanes 9 to 16). Sub-confluent BoMac and MDBK cells were either mock-infected (lanes 1 to 4 and 9 to 12) or infected with wt BFV-MDBK24 at a MOI of approximately 0.1 (lanes 5 to 8 and 13 to 16) and harvested 1, 2, 3, and 4 p.i. (as indicated below the blots). Identical amounts of protein were loaded and β-actin expression was monitored for proper protein loading (bottom panel). In this blot, the 75kDa splice variant of bovine ANKRD17 (top panel) was detected using the antiserum provided by T. Kufer and the full-length 40 kDa Bif1 protein was detected using the commercially available antiserum (middle panel). The bands specific for the 75 kDa ANKRD17 form and the 40 kDa Bif and 42 kDa β-actin are shown. Below the blots, the steady state protein levels were determined by densitometry, normalized to β-actin levels, and expression levels are displayed relative to the 1 d mock- and BFV-infected BoMac and MDBK cell values.

In a final experiment, the kinetics of miR-BF2-5p target gene expression/suppression during low-density BFV infection of BoMac and MDBK cells were analyzed. Cells were infected with wt BFV-MDBK24 (at a MOI of about 0.1) and mock-infected and harvested at 1, 2, 3, and 4 d p.i. for immunoblot analyses (Figure 10). Steady state levels of the BFV miRNA target proteins were determined by densitometry and normalized to β-actin levels. The changes in protein levels in this experiment relative to 1 d after mock- and BFV-infection of bovine BoMac and MDBK cells is shown in the table below the blots.

Importantly, in both bovine target cells, levels of ANKRD17 and, to a lesser degree, Bif1 increased over time in non-infected cells (lanes 1 to 4 and 9 to 12). These increases in steady state levels of both proteins were either strongly or even completely (ANKRD17) or only moderately (Bif1) abrogated in BFV-infected cells. The data indicate a complex relationship between cell growth and BFV infection-induced changes in target protein levels.

## 4. Discussion

The data presented here show that the miRNA cassette encoded by a distinct region of the U3 sequence of the BFV LTR is important for efficient virus replication in vitro in immortalized bovine cells. The deletion of the about 150 nt-long miRNA cassette in the U3 part of the LTR does not overlap with BFV proteins or BFV Tas response elements or other known cis-acting elements implicated in BFV replication [42,46]. The miRNA deletion can be almost completely complemented in vitro in trans by providing the major BFV miRNA, BF2-5p. This experiment strongly implies that the gross deletion of the miRNA cassette does not affect essential elements at a detectable level and that BF2-5p is of prime importance, at least in vitro, in immortalized bovine cells. This conclusion is also supported by in silico predictions of potential BFV miRNA targets in the bovine genome: for BF2-5p, a high number of high-score target genes was identified while only a few promising targets were found for the second-most abundant miRNA, BF1-5p, whilst the other BFV miRNA yielded no high-score targets.

In addition, the deletion of the complete miRNA cassette from the BFV genome did not detectably impair BFV Gag and Pol expression and processing as detected by analyzing transfected HEK293T and bovine MDBK cells and enriched BFV particles. Proper Gag and Pol processing in particles (and cell lysates) strongly argues for proper genome encapsidation since genome-mediated packaging of Pol into forming FV capsids is considered a necessary prerequisite for Gag and Pol processing by the FV protease [43]. The presence of uncleaved Pol proteins in the particle preparations of the in vitro selected BFV variant with high titer cell-free transmission phenotype may either reflect an impaired adaptation to this novel phenotype in this BFV variant independent of the presence or absence of the miRNA expression cassette. Alternatively, the selected BFV cell-free variants may correspond in this respect to PFV, where unprocessed Pol is also present in purified particle preparations dissimilar to the situation in feline FV (FFV), where unprocessed Pol is undetectable in enriched particle preparations [47,48].

For our proof of concept study on the function and relevance of the potential target genes, we had to focus on those bovine genes where reagents for immune-detection were available. In addition, we intentionally narrowed down to those genes with a known link to virus infection and replication and innate, intrinsic, and adaptive immunity in humans, mice, cattle, and other mammals. For these reasons, bovine Bif1 and ANKRD17 were subjected to in vitro studies on whether they are direct targets of the high-abundant BF2-5p miRNA. The data clearly confirm that both genes are in fact direct targets of this BFV miRNA in immortalized bovine cells. The BF2-5p miRNA effects on the overall levels of the target mRNAs was in both cases modest, but statistically significant. In contrast, Bif1 and ANKRD17 protein levels were substantially suppressed by the miRNA, either expressed from the BFV provirus, an U6 RNA Pol-III expression plasmid, or provided as an miRNA mimic compared to the corresponding controls lacking the miRNA cassette or the control mimic RNA sequence. The quantitatively different effects of the BF2-5p miRNA on target gene mRNA and protein levels are in line with the concept that miRNAs act mostly by suppressing protein translation [3,38].

ANKRD17 is implicated to have an important role in the control and regulation of NOD1 and NOD2 inflammatory immune signaling and thus the restriction and control of virus replication [20,21,22,23]. Aside from their function as direct pattern recognition receptors for bacterial peptidoglycans, NOD1 and NOD2 also act as signaling molecules involved in the regulation of inflammation and immunity [49]. ANKRD17 has been shown to physically interact with viral structural and non-structural proteins, however, the functional importance of these interactions is unknown [50,51]. Innate immunity appears to play an important role in controlling FV infections but its role in BFV is almost not understood [52,53,54,55]. Thus, potential downstream target genes of ANKRD17 were analyzed for their response toward BF2-5p miRNA expression. Analysis of the effect of BF2-5p on IFN-β and NF-κB RNA levels in vitro showed significant suppression of both potential target mRNAs. Extension of these miRNA studies by inclusion of other downstream targets, the analysis of mRNA and protein steady state levels as well as the effects on immune signaling in vivo and in vitro appears worth doing.

A link between Bif1 and virus replication is suggested by its function in autophagy, which may increase or decrease virus replication [56,57]. In PFV, autophagic flux is increased in infected cells and may result in reduced innate immune signaling [58,59]. However, PFV has suffered severe deletions in the U3 and may thus not or no longer be capable of expressing the authentic repertoire of miRNAs [60]. For time and resource reasons, functional studies have not been done for downstream targets of Bif1, but would be highly desirable in the future.

Due to the basic lack of reagents and a limited understanding of the bovine system compared to the much more intensively studied men or mice, several questions were not easy to study and have therefore not been addressed. For instance, the function(s) of ANKRD17 are currently not fully understood in humans and mice, and details on its function in innate immunity are scant. In addition, validated reporter constructs for immune signaling studies are rare or difficult to obtain. For bovines, no details on alternative splicing and alternative protein forms as described for human ANKRD17 are available. This situation, for instance, prevented trans-complementation studies to elucidate the mechanism of how ANKRD17 interferes with BFV replication. In addition, we do not have detailed information on the kinetics of innate (and adaptive) immune signaling in bovines, and therefore, we may have missed some of the downstream responses since we had simply sampled at the wrong time points. As shown here, the concentrations of ANKRD17 and Bif1 increased during cultivation of mock-treated cells. These increases are suppressed in BFV-infected cells during ongoing virus replication (Figure 10) and thus, steady-state protein levels of both BFV miRNA targets are probably the result of antagonistic effects on their steady-state levels.

Since the RNA Pol III-directed dumbbell-shaped pri-miRNAs cassettes of FVs represent a completely new way of miRNA biogenesis, conventional methods and reagents to study miRNA functions may or may not be appropriate for studying BFV miRNAs. For instance, the very high expression levels of individual BFV miRNAs and the pri-miRNA precursor in persistently infected cells in vitro are not known in other systems [8]. Thus, standard concentrations for transfected miRNA mimics, controls, and/or inhibitors may not be appropriate for BFV. To address this question, we determined whether increased amounts of transfected miRNA inhibitors further recover steady state levels of the identified target proteins in BFV-infected cells. As shown in Supplementary Figure S3, increased amounts of the co-transfected miRNA inhibitor did not further relieve protein suppression, but even appeared to increase suppression.

Finally, experimental inconsistencies and unexpected results related to the use and overexpression of mimic and inhibitor miRNAs may also be due to a “transcriptional override” in complex and currently unresolved regulatory networks [45]. Analyses in other bovine cell types including primary cells or ex vivo analyses would allow better definition of the BFV miRNA functions. The fact that the BFV Riems isolate used in this study was exclusively grown on bovine cells proficient in innate immunity may have, however, contributed to the success of this study. These and other points have to be taken carefully into account before starting future experiments.

Whether BFV miRNAs may have additional functions independent of the posttranscriptional control of cellular gene expression is currently unknown, but taking their high intracellular concentrations in chronically BFV-infected cells into account, this appears possible. The high intracellular concentrations of miR-BF1-5p and miR-BF2-5p [8] may be a prerequisite (or at least an advantage) for extracellular functions (e.g., in the form of free or exosome-associated miRNAs with local or systemic effects) [61]. For instance, it has recently been shown that under conditions of bacterial sepsis, the extracellular form of the murine and human miR-130b-3p is released into the blood and interacts, thereby attenuating the pro-inflammatory function of CIRP (cold-inducible RNA binding protein), a ligand of TLR4-mediated innate inflammatory signaling [62,63].

The data presented here are in line with the miRNA function of oncogenic BLV during in vitro and in vivo infections where deletion of the miRNAs did not abrogate virus replication [64,65]. Unfortunately, the BFV miRNA target genes predicted and validated in this study do not overlap with genes affected by BFV infection of BoMac in vitro, as published recently [55].

In summary, we demonstrate here for the first time that the FV miRNAs play a role during virus replication in vitro, however, more in vitro and especially in vivo studies are required to fully understand their functions for BFV biology and the underlying mechanisms used.

## Figures and Tables

**Table 1:** In silico prediction of bovine target gene potentially affected by BFV miRNAs miR-BF1-5p, miR-BF1–3p, and miR-BF2-5p using the miRNA target site prediction tools “miRanda–mirSVR” and “TargetScan”.

	miR-BF1-5p		miR-BF1-3p		miR-BF2-5p	
Target Rank	Target Score ^1^	Gene Symbol	Target Score ^1^	Gene Symbol	Target Score ^1^	Gene Symbol
1	80	BCL7A	62	TMEM135	100	ANKRD17
2	73	DUSP4	58	PHF20	99	Bif1 (SH3GLB1)
3	67	BCKDHB	58	BRWD1	99	AFAP
4	64	LYPD1	51	SUN1	98	AK3
5	64	RAD23A	51	DUSP6	96	FLRT3
6	62	ANKRD26	50	PRKCI	96	ARL6IP1
7	62	GAPVD1	41	FAM234B	96	ADAMTSL3
8	59	PPP1R13B	37	PI4K2B	96	PTER
9	59	OOSP2	35	USP38	96	BTBD7
10	58	ZNF701	33	UBE2G1	96	MON2
11	57	ZMYND11	29	ZFP62	96	UTRN
12	56	MKI67	28	COL4A1	96	GABRA1
13	56	GCC2	28	ABL2	96	DGKI
14	55	ATCAY	27	PPP2CA	95	CTDSPL2
15	54	RGPD5	25	STARD13	95	TTC26
16	54	RGPD8	25	CDK19	95	ITPKC
17	54	RGPD6	25	ELOVL4	95	BMP2K
18	52	NCCRP1	25	SEL1L	95	SLC7A1
19	52	HR	23	SNX18	95	DPY19L4
20	52	PAQR8	21	ERMP1	95	ZMYM2

^1^ Target scores are expressed as the mean values of the two algorithms used.

## Supplementary Materials

The following are available online at https://www.mdpi.com/1999-4915/12/11/1250/s1, Supplementary Figure S1: Deletion of the miRNA cassette does neither affect BFV Gag and Pol expression and processing nor BFV particle release and composition in infected MICL cells. Supplementary Figure S2: Specificity control for miR-BF2-5p mimic, negative control and inhibitor miRNAs. Supplementary Figure S3: No further increase in protein levels by increased amounts of inhibitor miRNAs in acutely BFV-infected cells.
