# Supplementary material for: Functional Analyses of Bovine Foamy Virus-Encoded miRNAs Reveal the Importance of a Defined miRNA for Virus Replication and Host–Virus Interaction
Source: Viruses. 2020 Nov 2;12(11):1250. doi: 10.3390/v12111250 (PMC7693620; doi:10.3390/v12111250)
Supplement: Supplementary file 1 [file viruses-12-01250-s001.zip › Wenhu2 suppl Fig1-new.pdf]

Suppl. Figure 1.

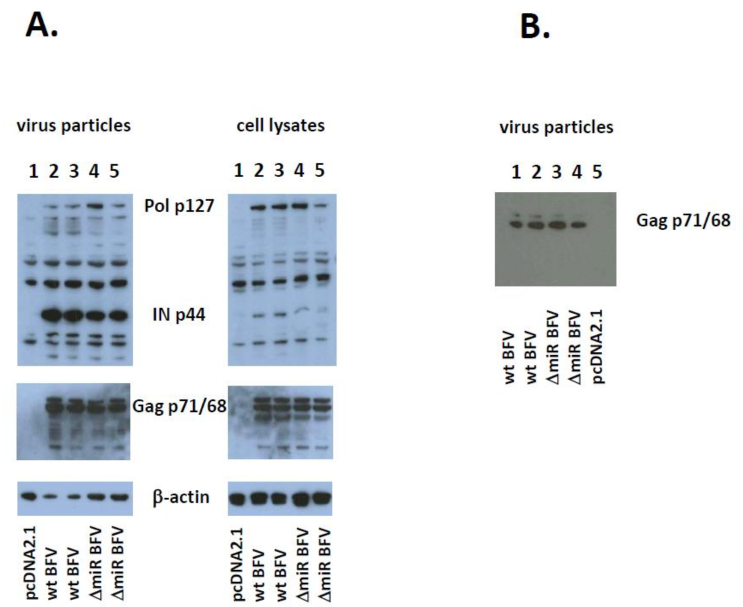

Suppl. Figure 1. Deletion of the miRNA cassette does neither affect BFV Gag and Pol expression and processing nor BFV particle release and composition in infected MICL cells (A) and transfected HEK293T cells. (A) Supernatants from HEK293T cells transfected with each two sub-clones of plasmids encoding high titer pCMV-BFV-MDBK 24 (wt BFV)and pCMV-BFV-MDBK 24-ΔmiRNA (ΔmiR BFV) and the pcDNA2.1 control plasmid and later co-cultured with MDBK cells (see legend to Fig. 2) were used to infect low-density MICL target cells at a MOI of 0.1. Five days after MICL cell infection and a comparable degree of infection, cells and BFV particles in the supernatants were harvested for BFV protein analyses. Each 15 μg of cell lysates and regular aliquots of enriched particles were subjected to immunoblotting (as given above the panels) using a cross-reactive rabbit anti PFV IN antiserum (top panels) and a pool of BFV-positive calf sera predominantly detecting BFV Gag proteins (middle panels). The BFV-specific proteins detected are labelled between the panels. A directly conjugated antibody against β-actin served as loading control (bottom panels). The detection of low amounts of b-actin in the enriched particle preparations may be due to an intentional overloading of the gel and the fact that the one-step centrifugation method is not sufficient to remove all contaminating proteins. The strong enrichment of the mature p44 IN protein in the particle preparations used indicates that the majority of proteins detected is derived from specifically released and thus processed BFV particles.

(B) Supernatants from HEK293T cells transfected with each two sub-clones of plasmids encoding high titer pCMV-BFV-MDBK 24 (wt BFV)and pCMV-BFV-MDBK 24-ΔmiRNA (ΔmiR BFV) and the pcDNA2.1 control plasmid were used to enrich released BFV particles for protein analyses. Regular aliquots of enriched particles were subjected to immunoblotting using a Gag rabbit hyper-immune serum. The BFV-specific Gag proteins are labelled.

The data show that particle budding as measured by immunoblotting of released particles is not obviously affected by deletion of the BFV miRNA cassette.
