# Supplementary material for: Functional Analyses of Bovine Foamy Virus-Encoded miRNAs Reveal the Importance of a Defined miRNA for Virus Replication and Host–Virus Interaction
Source: Viruses. 2020 Nov 2;12(11):1250. doi: 10.3390/v12111250 (PMC7693620; doi:10.3390/v12111250)
Supplement: Supplementary file 1 [file viruses-12-01250-s001.zip › Wenhu2 suppl Fig2 new.pdf]

Suppl. Figure S2.

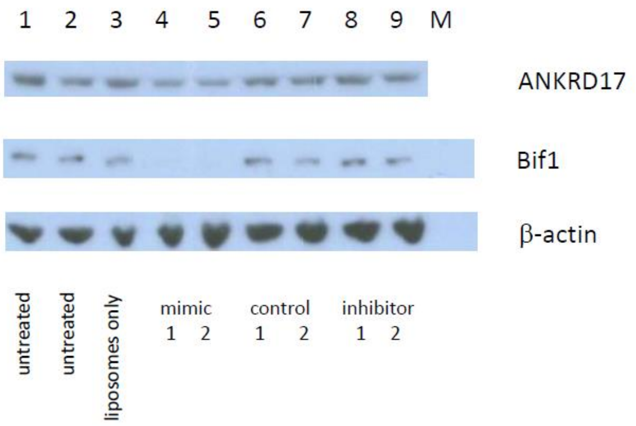

Suppl. Figure S2. Specificity control for miR-BF2-5p mimic, negative control and inhibitor miRNAs. Sub-confluent MDBK cells were transfected in duplicates in one experiment with standard amounts of the different miR-BF2-5p mimic, negative control and inhibitor miRNAs or with empty liposomes. Untreated MDBK cells served as an additional control as indicated below the blots. 3 d after transfection, cells were harvested for protein analyses. Each 15 μg of cell lysates were subjected to immunoblotting (as given above the panels) using the ANKRD17 antiserum provided by Prof. T. Kufer (top panel) and the commercially available Bif1 antiserum (middle panel). A directly conjugated antibody against β-actin served as loading control (bottom panel). The bands specific for the 75 kDa ANKRD17 form and the 40 kDa Bif and 42 kDa β-actin are shown.

The inhibitor miRNA induces clear suppression of the steady state levels of both target proteins, ANKRD17 and Bif1, while the inhibitor and the control miRNAs do not or only slightly affect steady state levels of both miR-BF2-5p target proteins.
