# Supplementary material for: Functional Analyses of Bovine Foamy Virus-Encoded miRNAs Reveal the Importance of a Defined miRNA for Virus Replication and Host–Virus Interaction
Source: Viruses. 2020 Nov 2;12(11):1250. doi: 10.3390/v12111250 (PMC7693620; doi:10.3390/v12111250)
Supplement: Supplementary file 1 [file viruses-12-01250-s001.zip › Wenhu2 suppl Fig3 new.pdf]

Suppl. Figure S3.

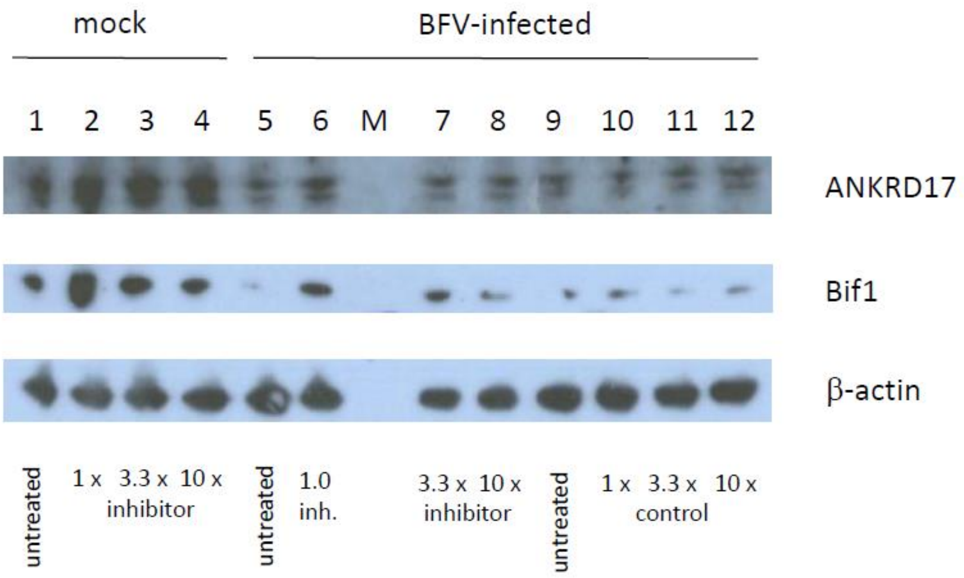

Suppl. Figure S3. No further increase in protein levels by increased amounts of inhibitor miRNAs in acutely BFV-infected cells. Sub-confluent MDBK cells were infected with BFV at a MOI of approximately 0.1 (lanes 5-12) or mock-infected (lanes 1-4) as given on top of the figure. One day p.i., cells were transfected with the miR-BF2-5p inhibitor and control at the standard concentration (1x; 25 pmol), and the 3.3- and 10-fold increased concentrations as given below the blots. Untreated MDBK cells served as controls. 3 d after transfection, cells were harvested for immunoblot analyses. Each 15 μg of cell lysates were subjected to immunoblotting using the ANKRD17 antiserum provided by Prof. T. Kufer (top panel) and the commercially available Bif1 antiserum (middle panel). A directly conjugated antibody against β-actin served as loading control (bottom panel). The bands specific for the 75 kDa ANKRD17 form and the 40 kDa Bif and 42 kDa β-actin are shown, the experiment was performed once.

At the standard concentration, the inhibitor of miR-BF2-5p partially rescued steady state levels of ANKRD17 and Bif1 while increased concentrations of the inhibitor did not further rescue protein levels but led even in part to lower protein levels. As expected, the control miRNA did not rescue steady state levels of ANKRD17 and Bif1 in BFV-infected cells.
